# HE4 level in ascites may assess the ovarian cancer chemotherapeutic effect

**DOI:** 10.1186/s13048-018-0402-3

**Published:** 2018-06-14

**Authors:** Duanyang Liu, Dan Kong, Jing Li, Lei Gao, Di Wu, Yu Liu, Weiwei Yang, Lei Zhang, Jiang Zhu, Xiaoming Jin

**Affiliations:** 1 Department of Pathology, Harbin Medical University, 157 Baojian Road, Nangang District, Harbin, China; 2 Department of Oncologic Gynecology, Harbin Medical University Cancer Hospital, Harbin, China; 3 Department of Gynecology, the First Affiliated Hospital of Harbin Medical University, Harbin, China; 4 Department of Orthopedics, the First Affiliated Hospital of Harbin Medical University, Harbin, China

**Keywords:** Ovarian cancer, Malignant ascites, HE4, CA125, Marker

## Abstract

**Background:**

The clinical treatment of ovarian cancer with ascites is problematic. The main reasons for treatment failure are the susceptibility to intraperitoneal metastasis and chemotherapeutic drug resistance. The purpose and significance of this study is to evaluate which marker might evaluate treatment efficacy and improve the cure rate.

**Results:**

This study compared a no chemotherapy group with a chemotherapy group regarding the determination of carbohydrate antigen 125 and human epididymis protein 4 in ovarian cancer ascitic supernatants and cross-analyzed routine serum carbohydrate antigen 125 levels. The level of human epididymis protein 4 in the ascites of the chemotherapy group was significantly lower than that of the no chemotherapy group (*p* < 0.001). Moreover, the expression of ascitic human epididymis protein 4 correlated positively with serum carbohydrate antigen 125 levels (*p* < 0.001). MDR was positive in 13 of the 30 samples (43.33%) in the chemotherapy group with highly expressed CA125.

**Conclusion:**

The level of human epididymis protein 4 in ovarian cancer ascites may reflect the therapeutic effect of ovarian cancer patients, and a high level of human epididymis protein 4 might predict chemoresistance and the possibility of ascites formation. The determination of the expression of human epididymis protein 4 alone or combined with carbohydrate antigen 125 levels in both serum and ascites in ovarian cancer patients with ascites may have important significance for guiding and improving the treatment regimen.

## Background

Cancerous or malignant ascites is a common complication of advanced stage ovarian cancer, gastric cancer, liver cancer, colon cancer and other malignant tumors [1]. For patients with high-grade serous ovarian carcinoma, the clinical manifestations are often relatively less obvious, and the main symptoms pertain to the gastrointestinal digestive system, including nausea, anorexia, early satiety, abdominal distension, bloating, pain, tenesmus, constipation, back pain and urinary frequency [2]. When associated with malignant pleural effusion, cough and dyspnea are common. Moreover, high-grade serous ovarian carcinoma has the highest incidence among ovarian carcinomas [3], most of which are at an advanced stage due to its relatively atypical clinical manifestations when treatment is sought and are also accompanied by ascites, which indicates a poor treatment effect. Clinically, in the presence of ascites, ovarian cancer has been considered one of the hallmarks of an advanced stage with poor prognosis; moreover, severe hypoproteinemia often supervenes after multiple treatments, which might seriously affect patient quality of life [4, 5]. Specifically, the routine clinical care of ovarian cancer patients with ascites formation is to drain the ascites and to administer a regimen of intraperitoneal chemotherapy plus intravenous chemotherapy. However, to date, the evaluation of therapeutic regimens, their therapeutic efficacy and the prognostic markers during the treatment course in ovarian cancer patients with ascites have not been reported. Thus, no published reports regarding the treatment effectiveness evaluation of ovarian cancer with ascites therapy exist, particularly concerning the markers in groups receiving and not receiving chemotherapy.

CA125 is a surface glycoprotein antigen associated with the differentiation of Müllerian ducts. It is an important tumor marker in the diagnosis of ovarian cancer and may be used to monitor the prognostic changes of the disease during treatment [6]. Clinically, CA125 is mostly used as a single marker to monitor the treatment effect. The human epididymis protein 4 (HE4) is a new type of serum marker for the diagnosis of ovarian cancer [7, 8]. Currently, HE4 is believed to have a higher diagnostic value than CA125 [9, 10], and their combination may significantly improve the diagnostic assessment of ovarian cancer [11, 12]. HE4 alone or combined with CA125 have recently been used as serum prognostic indicators for ovarian cancer patients. However, they are used in only some hospitals, and no reports concern their analysis in ovarian cancer malignant ascites. This study intends to determine these two types of markers in ovarian cancer ascites and to concurrently compare the expression of HE4 in ovarian cancer ascites with the serum CA125 levels of patients to evaluate the relationship between the level of HE4, prognosis, and drug resistance, particularly in patients with recurrent ascites formation to provide a basis for revising the treatment regimen at any time in ovarian cancer or ovarian cancer patients with ascites.

However, CA125 alone or combined with HE4 for ovarian cancer detection in hospitals mainly assessed ovarian cancer patients using serum tests. The CA125 or HE4 levels in ascites, the correlation of CA125 and HE4 in the ascites of ovarian cancer patients who received and did not receive chemotherapy, and the correlation of their levels with serum values have not been reported to date.

## Methods

### Major materials and reagents

In total, 130 cases of ovarian cancer with malignant ascites were obtained from the Department of Oncologic Gynecology of the Tumor Hospital Affiliated with Harbin Medical University from December 2013 to January 2016. Seventy-six of these patients were not administered chemotherapy, and 54 patients received chemotherapy. All of the cases had complete clinical pathological data: 76.93% (100/130) were the serous type, and the others included mucinous and some other types. Written informed consent was obtained from each patient, and the study was approved by the institutional ethics committee of Harbin Medical University.

ACK Lysis Buffer was purchased from Beijing Leagene Biotechnology Co., Ltd., Beijing, China. The CA125 polyclonal antibody and HE4 polyclonal antibody were purchased from Beijing Biosynthesis Biotechnology Co., Ltd. The CA125 ELISA kit was purchased from Beijing North Institute of Biological Technology Co., Ltd. The HE4 ELISA kit was purchased from Sweden Fujirebio Diagnostics, Inc. The flow cytometry antibody against CA125 was purchased from Biolegend.

### Methods

#### Preparations of ovarian cancer ascitic fluid

Ascites samples collected from patients diagnosed with epithelial ovarian carcinoma were centrifuged for 10 min at 3500 r.p.m. The supernatants were stored in 50-ml tube and 0.5-ml tube, respectively, at − 80 °C for further analysis. Following incubation for 10 min after the addition of ACK lysis buffer, the precipitates were centrifuged for 5 min at 2500 r.p.m. The supernatants were discarded, and then 2.5% glutaraldehyde or 10% formaldehyde for fixation was added to the white pellets separately for subsequent evaluation by electron microscopy or to prepare paraffin-embedded HE sections, respectively.

#### Pathologic assessment of ovarian cancer ascitic fluid-precipitated cell components

Samples for light microscopy: the cells were fixed with 10% formaldehyde for 24 h and processed into paraffin blocks routinely and then sectioned. Hematoxylin and eosin staining was performed on the sections.

Samples for electron microscopy: the cells were fixed for 2 h with 2.5% glutaraldehyde in PBS (pH 7.4). After rinsing with PBS, the tissues were fixed in 1% osmium tetroxide for 2 h at 4 °C, washed 3 times in distilled water for 5 min each time, dehydrated in a graded series of acetone (50%, 75%, 90%, 100%), and embedded in Epon 812. Ultrathin sections were cut into slices 50-nm to 70-nm thick, double-stained with uranyl acetate and lead citrate, and examined under an H-7650 electron microscope.

#### Flow cytometric analysis of ovarian cancerous ascites

The samples were prepared to determine the CA125 levels of the ascitic fluid. After cell counting, 1 × 10^6^ cells were added into each 1.5-ml EP tube, rinsed twice with PBS and centrifuged for 5 min at 1500 r.p.m. After a single rinse with staining buffer, the relevant antibody was added. After 20 min of incubation in a dark at room temperature on a shaker, the samples were rinsed twice with PBS, fixed with 500 μl of 1% paraformaldehyde, and then analyzed (repeated 3 times).

#### Immunohistochemical determination of CA125 and HE4 in ovarian cancer ascitic fluid centrifuged cells

The centrifuged cell pellets were processed into sections. CA125 and HE4 were added to the cells, which were then incubated overnight at 4 °C. The cells were treated with a secondary anti-rabbit antibody, incubated for 30 min at 37 °C, and then stained with DAB and counterstained with hematoxylin. Next, the cells were mounted on slides, and images were collected using a light microscope (Nikon E800 Multifunctional Biological Microscope; Image Acquisition Software Nikon ACT 21, version 6.1).

#### ELISA for HE4 in ovarian cancer ascitic supernatants after centrifugation

All the reagents and specimens were brought to room temperature before use. Next, calibrators B-F, controls 1 and 2, wash solution and tracer working solution were prepared. Each strip was washed once with wash solution, and 25 μl of each of the HE4 calibrators (CALA, B, C, D, E and F), HE4 controls (C1, C2) and unknown specimens (unk) were pipetted into the trip wells. Next, 100 μl of biotinylated anti-HE4 was added to each well using a 100-μl precision pipette, and the plates were incubated under constant agitation for 1 h (±10 min) at room temperature (20~ 25 °C). The supernatant was aspirated, and each strip was washed 3 times; 100 μl of tracer working solution was added to each well and the plates were then incubated with constant shaking for 1 h (±10 min) at room temperature (20~ 25 °C). The supernatant was aspirated, and each strip was washed 6 times before the addition of 100 μl of TMB HRP substrate to each well (added as quickly as possible; the time between the addition to the first and last well should not exceed 5 min). The plates were incubated for 30 min (±5 min) at room temperature (20~ 25 °C) with constant shaking and protection from direct sunlight. Next, 100 μl of stop solution was added, and the absorbance at 405 nm was read in a microplate spectrophotometer within 15 min after the addition of the stop solution.

#### ELISA for CA125 in ovarian cancer ascitic supernatants after centrifugation

All the reagents and specimens were brought to room temperature for 30 min before use, and 50 μl of the calibrators S1, S2, S3, S4, quality control specimen, and sample were pipetted into two wells each. To each well (except the blank well), 50 μl of an enzyme combination was added, the contents were mixed, adhesive mounting was added, and the samples were incubated at 37 °C in a wet box for 1 h. Next, the wells were emptied, rinsed 3 times with wash solution for 10 s, and then flapped on the absorbent paper. Then, 50 μl of developer A and B were added and the samples were incubated at 37 °C with protection from exposure to direct sunlight for 15 min. After 50 μl of stop solution was added, the absorbance at 405 nm was read in a microplate spectrophotometer within 10 min after addition of the stop solution. All the above steps were repeated twice, and the average value was recorded.

#### Statistical analysis

ANOVA (F-test) was used for the comparison between groups. The Spearman method was used for correlation analysis. A single comparison with paired or non-paired t-test was used. A two-tailed probability of *p* < 0.05 was considered statistically significant, and *p* < 0.01 showed a statistically significant difference. Prism GraphPad was used for mapping and curve fitting.

## Results

### Identification of precipitated cells from ovarian cancer ascites

**Fig. 1:**
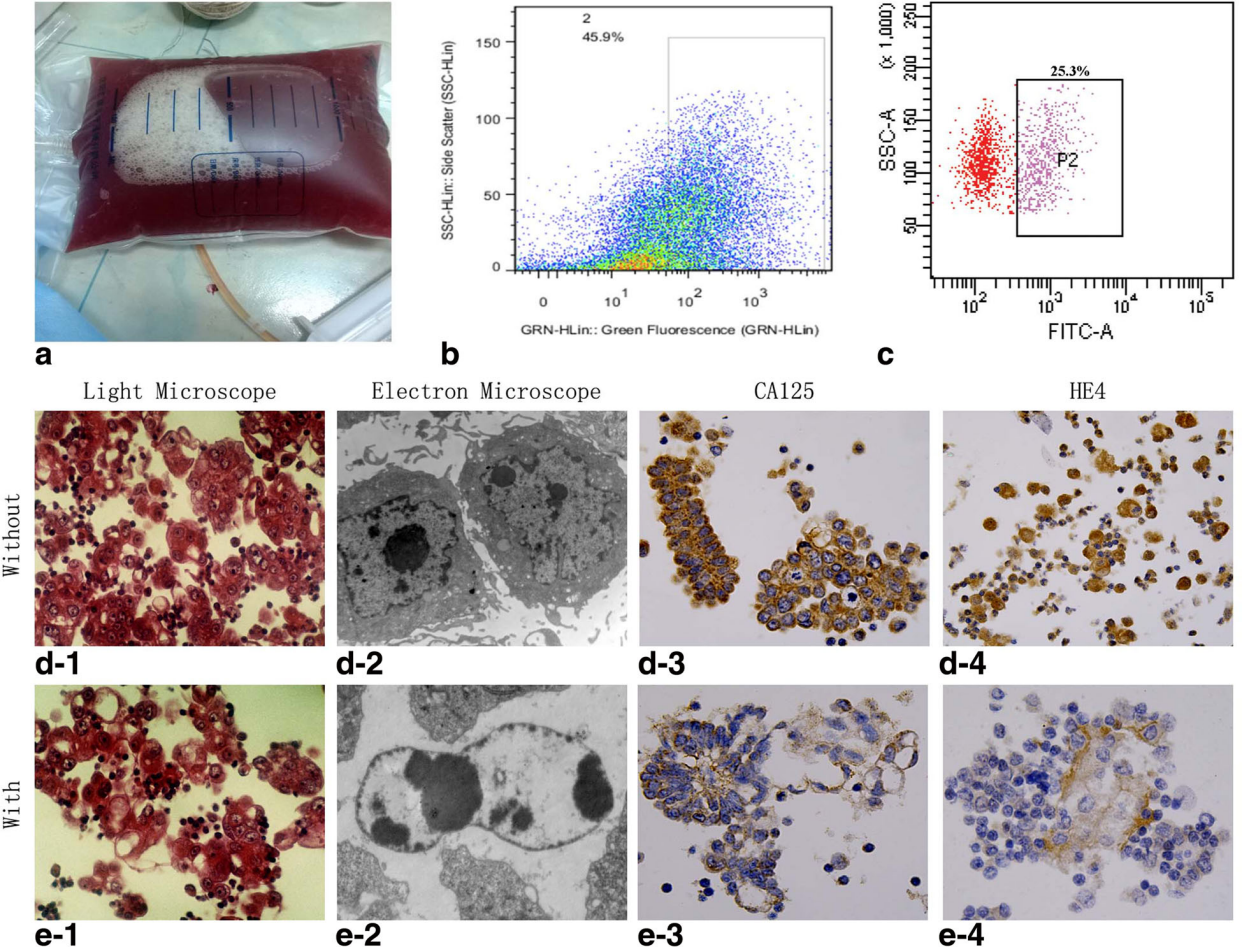
Identification of precipitated cells from ovarian cancer ascites. **a**. Bloody appearance of ovarian cancer malignant ascites; **b**. In the group without chemotherapy, the tumor cells occupied 45.9% of the field; **c**. In the group with chemotherapy, the tumor cells occupied 15.3% of the field; **d-1**. Tumor cells predominate under light microscopy, HE× 400; **d-2**. The nucleus is large with increased nucleosomes under electron microscopy, EM × 6000; **d-3**. The expression of CA125 in the tumor cells is high, sABC× 400; **d-4**. The expression of HE4 is high in the tumor cells, sABC× 400; **e-1**. The tumor cell number was decreased under light microscopy, and some of the tumor cells appeared vacuolated, HE× 400; **e-2**. Most of the tumor cells were apoptotic, EM × 6000; **e-3**. The expression of CA125 was low in the tumor cells, sABC× 400; **e-4**. The expression of HE4 was weak in the tumor cells, sABC× 400

**Fig. 2:**
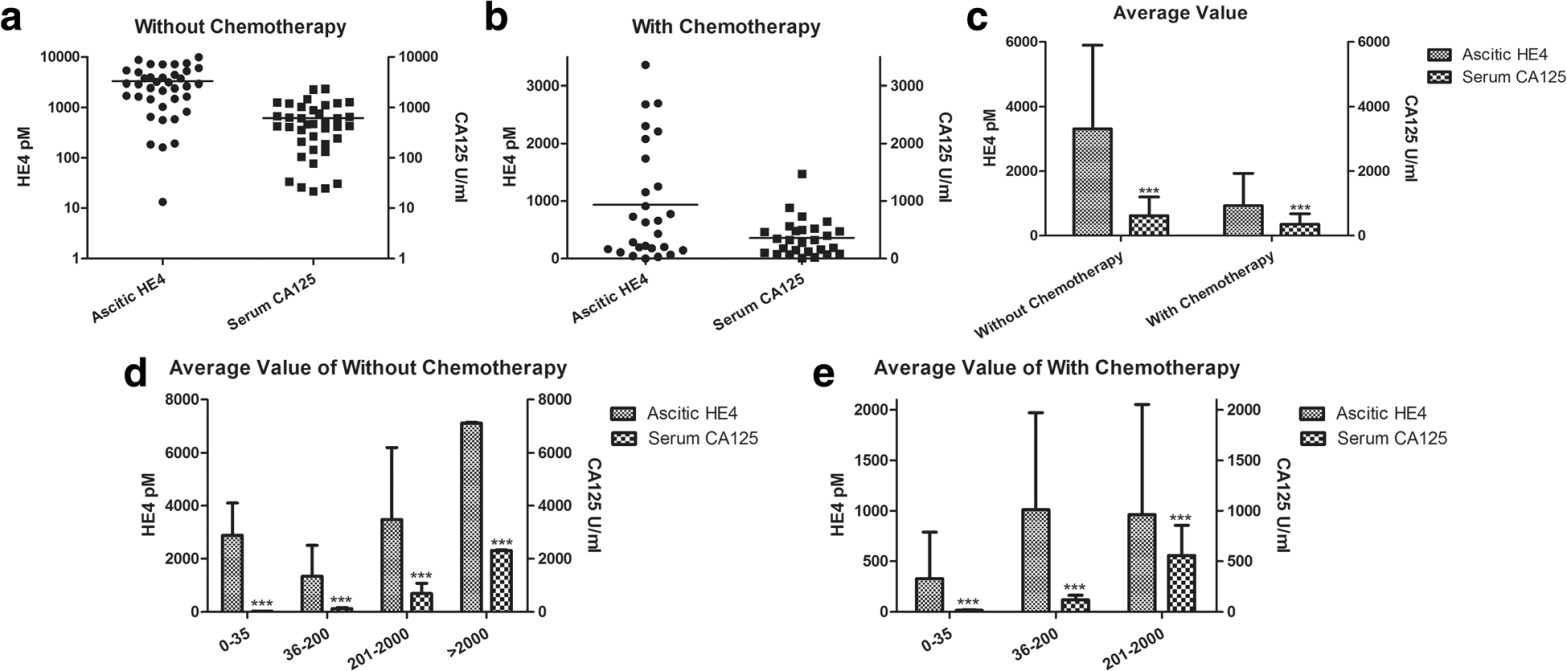
The relationship between the expression of ascitic HE4 and serum CA125 in ovarian cancer. **a**. The contents of ascitic HE4 and serum CA125 in the no chemotherapy group; **b**. The contents of ascitic HE4 and serum CA125 in the chemotherapy group; **c**. A comparison of the average values for ascitic HE4 and serum CA125 in the groups with and without chemotherapy (***p < 0.001); **d** & **e**. The comparison of ascitic HE4 and serum CA125 both in the groups with and without chemotherapy and according to the different categories of serum CA125 values; all the differences were significant (***p < 0.001)

**Fig. 3:**
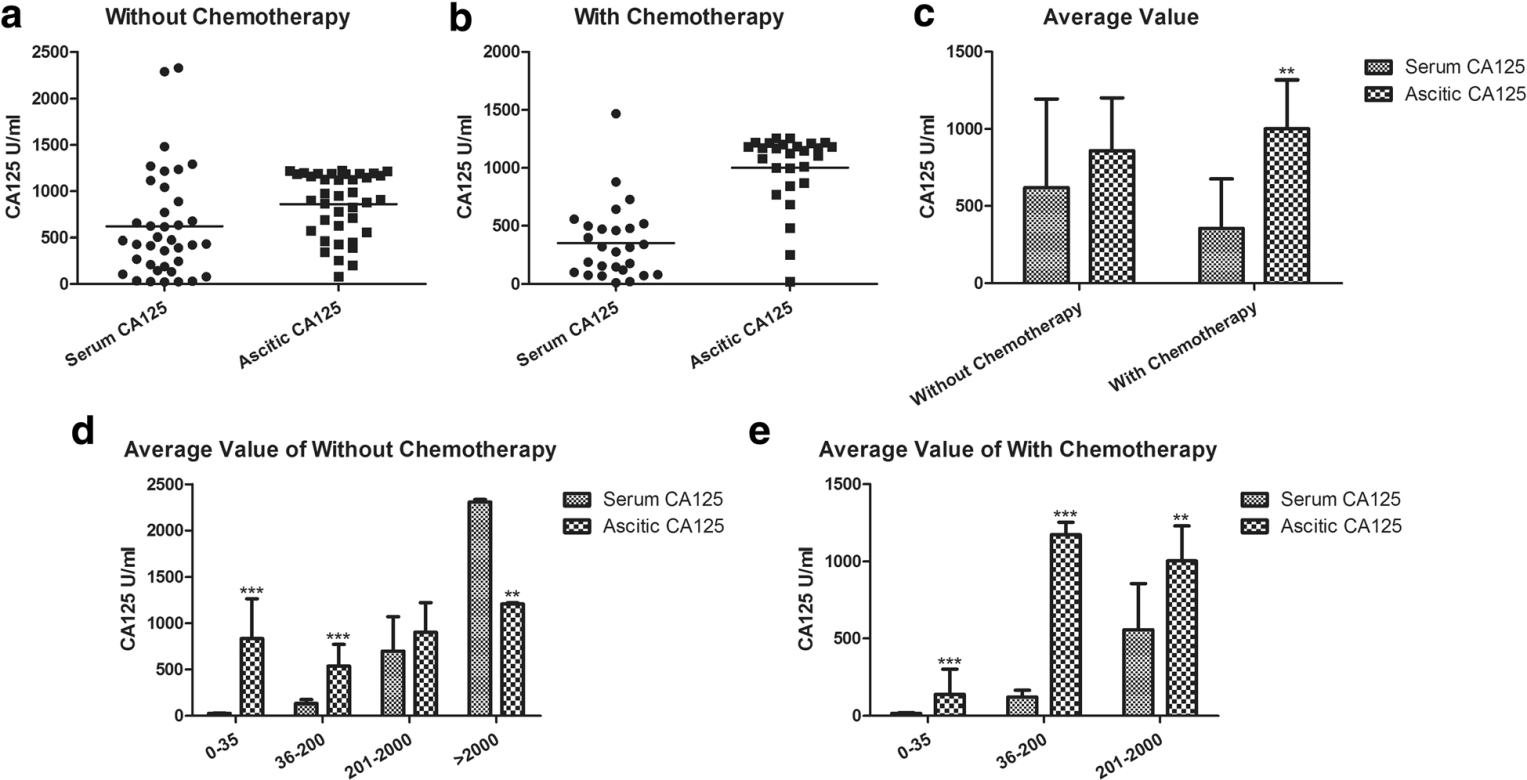
The relationship between the expression of serum and ascitic CA125 in ovarian cancer patients. **a**. The contents of serum and ascitic CA125 in the no chemotherapy group; **b**. The contents of serum and ascitic CA125 in the chemotherapy group; **c**. A comparison of the average values of serum and ascitic CA125 in the groups with and without chemotherapy (**p < 0.01); **d** & **e**. The comparison of serum and ascitic CA125 both in the groups with and without chemotherapy and according to the different categories of serum CA125 values (**p < 0.01, ***p < 0.001)

**Fig. 4:**
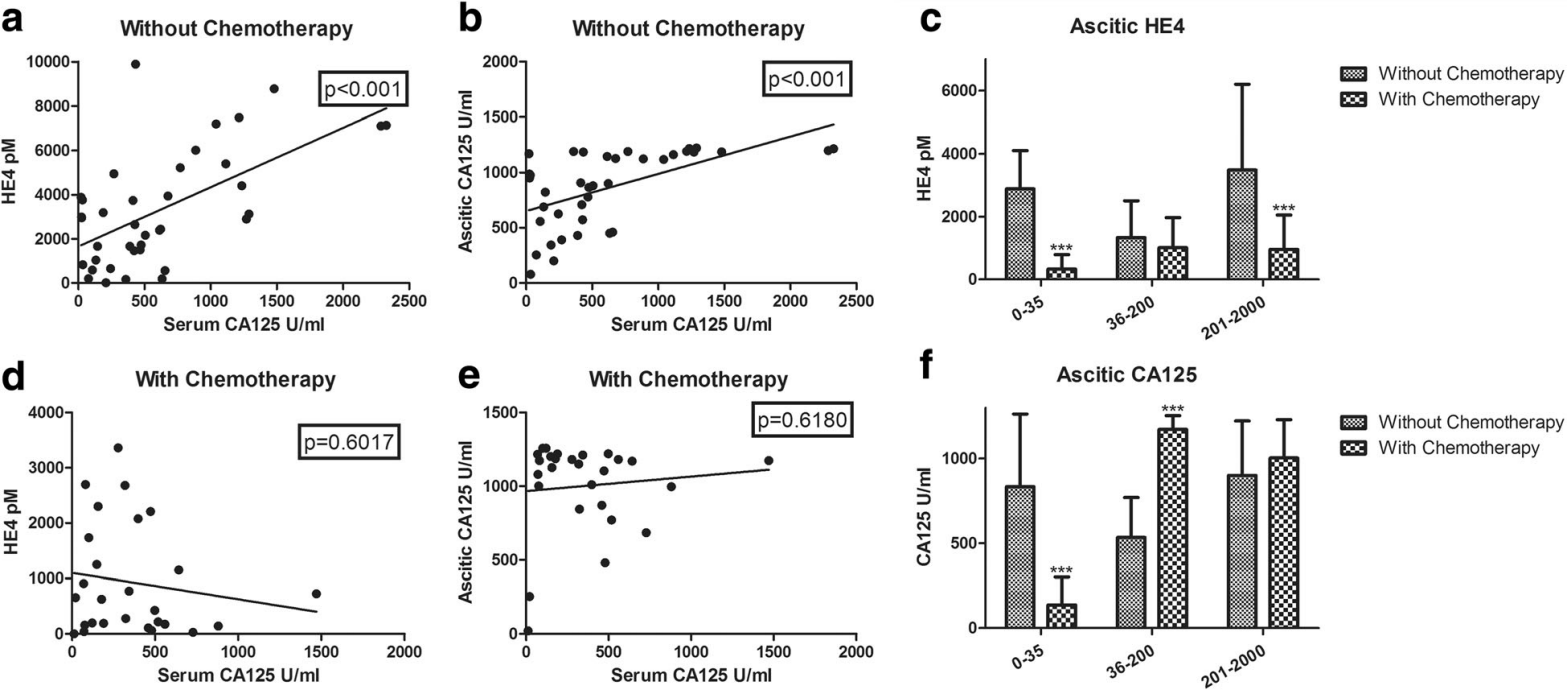
Correlations of CA125 and HE4 in ovarian cancerous ascitic supernatant compared with serum CA125. **a**. Serum CA125 positively correlated with ascitic HE4 in the no chemotherapy group and the difference was significant (***p < 0.001); **b**. Serum CA125 positively correlated with ascitic CA125 in the no chemotherapy group and the difference was significant (***p < 0.001); **c**. Relationships of ascitic supernatant HE4 and serum CA125 in the different categories (***p < 0.001); **d** & **e**. The correlations of serum CA125 compared with ascitic HE4: ascitic CA125 levels did not differ significantly (*p* > 0.05) in the chemotherapy group; 4 **f**. Relationships of ascitic supernatant CA125 and serum CA125 levels among the different categories (***p < 0.001).s

Most of the hospital-acquired ascitic fluid samples were bloody (Fig. 1a). Flow cytometry showed that the tumor cell content of the ascites from patients who did not receive chemotherapy was 45.9% (Fig. 1b), and 25.3% from those who received chemotherapy (Fig. 1c). In the no chemotherapy group, the tumor cells were diffusely arranged or cloudy gathered and connected with each other under light microscopy (Fig. 1d-1), whereas under electron microscopy the nuclei were large, rich in nucleosomes and with intrusions on the nuclear membrane (Fig. 1d-2). The expression of CA125 was evenly distributed in all tumor cells’ cytoplasm or membrane (Fig. 1d-3), whereas HE4 was highly expressed in a granular pattern within the cytoplasm of the tumor cells (Fig. 1d-4). In the chemotherapy group, the tumor cells were quantitatively decreased under light microscopy, and the cytoplasm of the tumor cells was focally vacuolated (Fig. 1e-1). Most tumor cells appeared apoptotic under electron microscopy (Fig. 1e-2). The expression of CA125 was low (Fig. 1e-3), and the expression of HE4 was reduced in the tumor cells (Fig. 1e-4).

### Comparison of ovarian cancer ascitic supernatant HE4 and serum CA125 levels

All 130 specimens were divided into 4 groups according to the level of serum CA125 in both the chemotherapy and no chemotherapy groups (Table 1). The expression of HE4 in the supernatant of ovarian cancer ascites differed significantly (Fig. 2a-2c) compared with the average value of total serum CA125 between the chemotherapy group and the no chemotherapy group; additionally, this expression also differed significantly (Fig. 2d, e) among the different levels of serum CA125.

**Table 1:** The category of serum CA125 of all the 130 cases

CA125 U/ml	Without Chemotherapy (*n* = 76)	With Chemotherapy (*n* = 54)
0–35	10	4
36–200	10	20
201–2000	52	30
> 2000	4	0

### Comparison of CA125 levels between ascites and serum from ovarian cancer patients

According to the different serum CA125 levels, the ascitic CA125 levels were compared. While no significant difference of serum CA125 was observed in group C without chemotherapy, all the other groups showed significant differences (Fig. 3a-e). As shown in Figure 2d, ascitic HE4 differed significantly compared with serum CA125 in the same no chemotherapy group C, which indicated that HE4 was more sensitive than CA125.

### Correlation of CA125 and HE4 in ovarian cancerous ascitic supernatant with serum CA125

The expression of both HE4 and CA125 from ovarian cancer ascites supernatant correlated positively with serum CA125 in the no chemotherapy group, and the differences were significant (*p* < 0.001, Fig. 4a, b); however, the differences were not significant in the chemotherapy group (*p* > 0.05, Fig. 4d, e). The expression of HE4 in the ascitic supernatant from the groups with and without chemotherapy differed significantly (*p* < 0.001) in group A and group C, although no significant difference was observed for group B (*p* > 0.05, Fig. 4c). The CA125 expression in ovarian cancer ascites differed significantly between group A and group B (*p* < 0.001), with no significant difference for group C (*p* > 0.05, Fig. 4f). Investigation of the clinical medical records revealed that MDR was positive in 13 of the 30 samples (43.33%) in group C with chemotherapy, (Table 1) and that the ascites recurred within 6 months after the treatment.

### Analysis of CA125 and HE4 with clinicopathological data in 130 cases of ovarian cancerous ascitic supernatant

The expression of HE4 in ascites and the serum CA125 level differed significantly between the chemotherapy group and the no chemotherapy group (*p* < 0.001). The expression of HE4 in the ascites of the serous ovarian cancer group was low (*p* < 0.001), particularly among high-grade serous carcinoma patients. Additionally, the expression of serum CA125 in the group with lymph node metastasis was high (*p* < 0.05); however, no significant differences were observed regarding other clinicopathological data (Table 2). CA125 and HE4 mainly correlated with whether the patients had undergone chemotherapy, the presence of metastasis, and the histological type, particularly for high-grade serous carcinoma.

**Table 2:** Correlation analysis of CA125 and HE4 with clinical pathological data

	Cases *n* = 130(%)	IH Detection (%)		Average Value		
		CA125	HE4	Serum CA125	Ascitic HE4	Ascitic CA125
Age						
≤ 50	26(20.00)	18(69.23)	18(69.23)	273.23	3153.48	952.93
>50	104(80.00)	88(84.62)	88(84.62)	568.07	2116.72	910.46
Chemotherapy						
With	54(41.54)	46(85.19)	46(85.19)	354.71***	932.56***	1002.04
Without	76(58.46)	60(78.95)	60(78.95)	618.8	3312.78	859.92
Histological type						
Serous	100(76.93)	94(94.00)	94(94.00)	554.23	2133.79***	961.44
LGSC	3(3.00)	1(1.06)	1(1.06)	612.50	2388.38	1146.59
HGSC	97(97.00))	93(98.94)	93(98.94)	552.43	2125.92***	955.71
Mucinous	10(7.69)	2(10.00)	2(10.00)	27.34	2881.84	732.97
Others	20(15.38)	10(50.00)	10(50.00)	524.34	2996.59	799.52
LN metastasis						
With	62(47.69)	56(90.320)	56(90.32)	632.56	2220.1	910.17
Without	68(53.31)	50(73.53)	50(73.53)	396.54*	2418.87	926.96

*p < 0.05, ***p < 0.001

## Discussion

Malignant ascites is caused by primary or metastatic tumors of and to the peritoneum. Its presence is a prominent and direct clinical manifestation of tumor cell invasion and metastasis to the peritoneum. The main cause of malignant ascites is metastatic tumor; in older men, such occurrences are primarily caused by hepatic and gastrointestinal cancer, whereas in women, they are often caused by ovarian tumors and rare tumors such as mesenteric tumors, abdominal lymphoma, and malignant peritoneal mesothelioma [4, 5, 13]. Malignant ascites in patients with cancer is a clinical challenge. Routine laboratory examination of malignant ascites does not aid in its diagnosis; however, certain biochemical tests to diagnose cancerous ascites have drawn some attention. Current markers used for the diagnosis of ovarian cancer include carbohydrate antigen125 (CA125), human epididymis protein 4 (HE4), human lysophosphatidic acid (LPA), soluble mesothelin-related proteins (SMRP), the human bradykinin-releasing enzyme family (Hk), and osteopontin (OPN) [14]. Among these, CA125 is the most commonly used marker and is typically used for blood serum and tissue identification of ovarian cancer patients.

CA125 is a macromolecule glycoprotein secreted by embryonic epithelial cells. While it is not or only slightly secreted under normal conditions, in ovarian malignant lesions, the levels are significantly increased, which may be used for the diagnosis of epithelial ovarian cancer; currently, it is the first auxiliary diagnostic marker of ovarian cancer. Indeed, CA125 is the most widely used serum marker for ovarian cancer presently, and it is recognized for the monitoring of therapeutic effect and recurrence of ovarian cancer [15]. However, some investigators have observed that while the sensitivity of CA125 is high for the detection of ovarian cancer, the specificity is poor because it also may be highly expressed in many other malignant tumors [16–18]. Therefore, for the diagnosis of ovarian cancer, CA125 has some false-positivity. This study found that the average serum level of CA125 among ovarian cancer patients who received chemotherapy was decreased, which is widely recognized as one of the prognosis indicators for ovarian cancer. Whether CA125 may be used to assess the evaluation of ascites from ovarian cancer patients who received and those who did not receive chemotherapy has not been specifically reported. The expression of CA125 in ovarian cancer ascites was lower in the chemotherapy group than in the no chemotherapy group; however, no significant differences were observed. Moreover, in some individual cases, these levels were higher than those of the no chemotherapy group. The cause of continued ascites after chemotherapy, particularly multiple recurrent episodes, may be associated with chemotherapy drug resistance [19], a topic that warrants further investigation. In conclusion, some differences were observed in individual cases regarding the expression of CA125 in ovarian cancer ascites, indicating that it may not be reliably used to evaluate the treatment efficacy of ovarian cancerous ascites in patients who undergo chemotherapy.

Human epididymis protein 4 is a newly proposed tumor marker. It was first discovered by Kirchhoff [20] in 1991, using cDNA cloned from the HE4 gene of human distal epididymis epithelial cells that encode and secrete HE4 protein. HE4 is a new and promising ovarian cancer marker whose application prospects for early diagnosis and disease surveillance appear promising [21]. Some scientists found that HE4 was significantly associated with residual tumor size and operative time [22–24]; high HE4 levels represented an independent prognostic factor for worse prognosis and shorter OS, disease-free survival (DFS) and PFS [7, 25]. The median OS in patients with an HE4 change > 80% was significantly longer than that of those who underwent an HE4 change < 80% during neo-adjuvant chemotherapy (NACT) [26]. Multiple studies have indicated that HE4 might be used as a prognostic marker. Additionally, it might be used as a marker to detect other cancers [27–29]. Under normal physiological conditions, the expression level of HE4 is quite low, but it is high in ovarian cancer tissues and in those patients’ sera [30, 31]. Most investigators also found that serum HE4 values were significantly increased compared with those for CA125 [32–34] in ovarian cancer patients. Our study found that the recurrence of ascites from ovarian cancer patients might be inhibited after chemotherapy, including in patients who recurred, in whom both the total amount of ascites and ascitic HE4 levels were significantly lower than those values of the no chemotherapy group. The expression of HE4 in the no chemotherapy group was high and was low in the chemotherapy group, and the differences were significant. In some cases in which the CA125 difference was not significant, the difference in the HE4 level was significant, indicating HE4 is more sensitive. Some reports stated that the change in HE4 was more closely related to the therapy response and recurrence than the change of CA125, and the reduction of HE4 (63.3%) was more significant than that of CA125 [35]. Additionally, HE4 better correlated with the radiologic response than did CA125 in the neo-adjuvant chemotherapy group [36].

Currently, some hospitals have begun to use HE4 as another marker of ovarian cancer or have combined it with CA125 for monitoring and testing, although only as determined from the serum of ovarian cancer patients and not from ascites. The clinical symptoms in the vast majority of early ovarian cancer patients are not typical, and they more often present with digestive symptoms, including nausea, anorexia, early satiety, and other symptoms, which might delay early treatment and lead to tumor progression. Typically, patients present for examination and treatment after the development of ascites, and ascites is the main reason for them to be diagnosed and treated in many cases of high-grade serous ovarian carcinoma. Because ascites is in direct contact with primary tumors and because large amounts of tumor cells are present in situ in the ascites, the examination of markers in ascites may directly and effectively reflect tumor changes compared with serum analysis. Determining the values of CA125 and HE4 in ascites, which is discarded with the relief of the patients’ symptoms, might be promising. Additionally, these values might be used to evaluate the therapeutic effect and disease progression during further treatment. In the subsequent treatment process, the increased levels of HE4 and CA125 may be used as markers of chemotherapy resistance, and the determination of HE4 and CA125 levels in ascites might also assess whether patients at the time have developed resistance to disease, which might prompt an earlier treatment effect evaluation and do so more directly and effectively. At the time of this manuscript submission, no reports on the detection of HE4 in ovarian cancer ascites exist in China; indeed, in most hospitals, extensive measurement of HE4 has not been conducted.

The combined testing for HE4 and CA125 may have particular clinical significance. Some studies reported that the combination use of HE4 and CA125 might predict the surgical outcome, with a highly predictive impact on PFS and OS than for the markers individually [25, 37, 38]. Moreover, the combination of elevated levels of CA125 and HE4 were associated with significantly worse estimated median PFS with an HR 8.14 [38]. Therefore, HE4 may be used as an important marker in the diagnosis and treatment of ovarian cancer ascites. Previous studies showed increased expression of HE4 in the serum and cancerous tissues of ovarian cancer patients with chemotherapy resistance, and the progression-free survival and overall survival rates were reduced [25, 39, 40]. Our study also found that some malignant ascites patients continued to develop ascites during chemotherapy, with increased expression of ascitic HE4, suggesting that these patients possibly developed chemotherapy resistance. After chemotherapy, the values of CA125 and HE4 in the serum and ascites of ovarian cancer patients will decrease, and chemotherapy resistance accounted for those with abnormal high values. Subsequently, our team compared factors in the chemotherapy-sensitive tumors to those of the chemotherapy-resistant tumors, we found that the levels of CA125 and MDR increased significantly, while the level of apoptosis decreased and the level of autophagy increased. We concluded that increased autophagy and inhibited apoptosis might lead to drug resistance in ovarian cancer. The application of an autophagy inhibitor combined with chemotherapeutic drugs is expected to improve the chemosensitivity of ovarian cancer patients (the Journal of Cell Death and Disease has officially accepted the manuscript) [41]. The evidence in this area requires further investigation.

In summary, the preferred treatment of ovarian cancer with ascites is chemotherapy, and the screening of the chemotherapeutic effect evaluation index is crucial. The expression of HE4 in ovarian cancer ascites combined with the level of serum CA125 in ovarian cancer patients may directly reflect the treatment effectiveness. This finding may provide significant guidance and aid clinicians to assess the prognosis and to modify the treatment.

## Conclusions

The level of human epididymis protein 4 in ovarian cancer ascites may reflect the therapeutic effect of ovarian cancer patients, and a high level of human epididymis protein 4 might predict chemoresistance and the possibility of ascites formation. The determination of the expression of human epididymis protein 4 alone or combined with carbohydrate antigen 125 levels in both serum and ascites in ovarian cancer patients with ascites may have important significance for guiding and improving the treatment regimen.

## Abbreviations

ACK: Ammonia-chloride-potassium
ANOVA: Analysis of variance
CA125: Carbohydrate antigen 125
DAB: Diaminobenzidine
DFS: Disease-free survival
ELISA: Enzyme-linked immunosorbent assay
EP: Eppendorf
Fig: Figure
HE4: Human epididymis protein 4
HGSC: High-grade serous carcinoma
HR: Hazard ratio
HRP: Horseradish peroxidase
LGSC: Low-grade serous carcinoma
LPA: Lysophosphatidic acid
NCAT: Neo-adjuvant chemotherapy
OPN: Osteopontin
OS: Overall survival
PBS: Phosphate-buffered solution
PFS: Progression-free survival
pH: Hydrogen ion concentration
r.p.m.: Revolutions per minute
SMRP: Soluble mesothelin-related proteins
TMB: Tetramethylbenzidine
UNK: Unknown
